# Metaphor Processing Dysfunctions in Schizophrenia Patients With and Without Substance Use Disorders

**DOI:** 10.3389/fpsyt.2020.00331

**Published:** 2020-04-24

**Authors:** Ewa Karabanowicz, Ernest Tyburski, Karol Karasiewicz, Andrzej Sokołowski, Monika Mak, Monika Folkierska-Żukowska, Wioletta Radziwiłłowicz

**Affiliations:** ^1^Institute of Psychology, University of Szczecin, Szczecin, Poland; ^2^Institute of Psychology, SWPS University of Social Sciences and Humanities, Poznan, Poland; ^3^Department of Neurology, Memory and Aging Center, UCSF Weill Institute for Neurosciences, University of California, San Francisco, San Francisco, CA, United States; ^4^Independent Clinical Psychology Unit, Pomeranian Medical University, Szczecin, Poland; ^5^Interdisciplinary Centre for Behavioural Genetics Research, Faculty of Psychology, University of Warsaw, Warsaw, Poland; ^6^Institute of Psychology, University of Gdansk, Gdansk, Poland

**Keywords:** metaphor processing, cognitive functions, schizophrenia, substance use disorder, dual diagnosis

## Abstract

**Background:**

Patients with schizophrenia have difficulties comprehending metaphors, which significantly impedes communication. However, this topic has not been thoroughly studied in people with a dual diagnosis. On this basis, we formulated two research aims: a) to compare the ability to comprehend metaphors in schizophrenia patients without (SZ) and with substance use disorder (SZ-SUD) and b) to determine the relationship between the processing of metaphorical content and the severity of psychopathological symptoms in both clinical groups.

**Methods:**

A total of 40 individuals with SZ and 40 individuals with SZ-SUD took part in the study. The control group was composed of 40 individuals without a psychiatric or neurological diagnosis. Four subtests from the Right Hemisphere Language Battery (Picture Metaphor Test, Written Metaphor Test, Picture Metaphor Explanation Test, Written Metaphor Explanation Test) were used to measure the ability to understand and explain metaphors.

**Results:**

Both groups of individuals with schizophrenia (SZ and SZ-SUD) scored lower than individuals from the control group on all tests of metaphor processing. However, no differences were observed between the two clinical groups. SZ-SUD patients had better results for Picture Metaphor Explanation than for Written Metaphor Explanation. Negative symptoms were found to be significant predictors of difficulties with understanding and explaining metaphors.

**Conclusion:**

Individuals with schizophrenia, regardless of their substance use disorder (SUD) status, exhibit impaired metaphorical content processing. SUD in schizophrenia is not associated with significant impairments in understanding and explaining metaphorical content. Moreover, impairments in processing metaphorical content are associated with more severe negative symptoms of schizophrenia.

## Introduction

Metaphors are a form of non-literal language use. They are a special type of figurative expression whose meaning is generated by the semantic overlap between two distant concepts. In particular, conventional metaphors are used in everyday language and are embedded in the associated culture [e.g., *Marriage is a jail*; (1)]. They are stored in one's memory and processing them requires recollection of meaning rather than construction of meaning. This processing is a high-level language skill (2, 3). Metaphors are useful in all types of communication and context can be important for comprehension. One needs to comprehend metaphors to understand the intent of the speaker—sentence meaning is the simple interpretation of an utterance derived from its linguistic content and grammatical construction, whereas speaker meaning is deduced from the intention of the speaker (4). Processing metaphors requires the skills of understanding language, making inferences, abstraction, spotting analogies between phrases, knowledge of pragmatic rules, as well as recognizing the mental states of other people (5); thus, processing metaphors not only requires knowledge of semantic and syntactic rules, but also non-linguistic skills (6).

Individuals with schizophrenia (SZ) exhibit impairments in their cognitive, linguistic, and communication functions—inter alia, with expressing themselves adequately in a given situation, taking into account the knowledge and attitude of the listener, understanding their intention, and processing metaphors (7–11). Processing figurative content requires going beyond its literal meaning. Analyzing literal content without taking into account alternative metaphorical meanings may result in an inadequate understanding of an utterance in a given context. SZ patients have difficulty going beyond literal meanings; they give bizarre or idiosyncratic interpretations when asked to paraphrase figurative expressions (1).

SZ patients have more difficulty understanding conventional metaphors than do healthy individuals (1, 12–14); they exhibit difficulty giving verbal explanations of metaphors and make a significantly higher number of literal incorrect (giving the literal meaning of words) and abstract incorrect (giving an answer that is abstract, but not in line with the meaning of the metaphor) errors than individuals without SZ (15). A study by Mossaheb et al. (16) showed that processing metaphors may be related to processing speed, cognitive flexibility, and a range of intelligence quotient (IQ) subtests. However, other studies show that low IQ does not explain the deficits in comprehension of metaphors and irony exhibited by SZ patients (6, 17). It has been shown that even when IQ, years of education, and capacity for theory of mind and associative learning are factored in as covariates, SZ patients still give significantly more incorrect answers on metaphor tasks (18). Moreover, impairments in the recognition and paraphrasing of conventional metaphors and the generation of novel metaphors may be related to negative symptoms (16).

Additionally, the decreased ability of individuals with SZ to understand figurative content is associated with changes in the brain, which has been shown by research using blood-oxygen-level-dependent (BOLD) imaging in functional magnetic resonance studies. Mashal et al (19) suggested that the processing of metaphors in schizophrenia involves compensatory recruitment of the left, middle, and inferior temporal gyrus and left precuneus. Moreover, another study (13) showed significant correlation between activation in the right precuneus (i.e., superior parietal lobule) and activation in the right posterior superior temporal sulcus during processing of conventional metaphors in schizophrenia patients.

Substance use disorder (SUD) often co-occurs in patients with schizophrenia (in up to 50% of cases), which is referred to as a dual diagnosis. Some studies show that patients with SUD have difficulties with emotional processing [e.g., expressing emotions and emotion recognition; (20–22)]. These functions are associated with understanding conventional metaphors, so difficulties with emotional processing may be associated with diminished ability to process conventional metaphors (23). Additionally, general knowledge (24) and executive functions (25) play an important role in metaphor processing. The meaning of conventional metaphors is stored in an individuals' mental lexicon as a unitary representation. It is possible that executive functions and this mental lexicon could be altered by SZ (26). This alteration could be greater the longer the duration of illness and/or in the case of SUD comorbidity.

One of the mechanisms which can cause impairment of metaphor comprehension are the neurotoxic effects of SUD on the central nervous system (27, 28). Changes also occur in SUD patients as time progresses—drug intake is reinforced by increased dopaminergic activity, leading to neurotoxic damage in fronto-subcortical circuits in the long-term and exacerbating the pre-existing dopamine deregulation in SZ patients (29). People with heroin, cocaine, and alcohol use disorders have been found to have decreased volumes in areas of the frontal cortex that are involved in higher-order cognition (30, 31). Drug use disorder is also associated with morphological changes in dendrites and dendritic spines in the prefrontal cortex (32). Blood flow and metabolism in the prefrontal cortex is impaired in individuals with alcohol use disorder, which leads to cognitive dysfunction and structural changes in the brain (33). Network dysregulation in cortical and temporal limbic in schizophrenia may act similarly to the network dysregulation which occurs in drug use disorder (34). A new unifying hypothesis has been proposed that combines recent evidence from epidemiological and genetic studies with brain imaging and pre-clinical studies to provide an updated formulation of the basis of substance use in SZ patients. It suggests that the genetic determinants of risk of schizophrenia (especially those pertaining to neural systems that contribute to the risk of both psychosis and SUD) make patients vulnerable to substance use (35). It is worth noting that some studies have found no differences in gray matter in the prefrontal cortex between SZ patients and participants with SUD (36, 37).

Individuals with schizophrenia and substance use disorder (SZ-SUD) are usually excluded from studies, which is why there is limited knowledge about cognitive functions in individuals with dual diagnoses (38). As far as we know, the ability of SZ-SUD patients to comprehend and explain metaphors has not yet been studied. Some reports suggest that such patients present deficits in memory, learning abilities, decreased visuospatial skills and executive functions, impaired psychomotor abilities, and difficulties with decision making (28, 39). Because of the relationship of cognitive processes and executive functions with metaphor comprehension (1), it is possible that individuals with dual diagnoses are characterized by even greater deficits in understanding metaphorical content. SUD may lead to earlier onset of schizophrenia (40), aggravation of the disease, more severe course of the disease, and an increased number of hospitalizations (41), which, as a consequence, harms the patient and the efficiency of their cognitive functions. Moreover, the treatment of dual diagnosis patients can be more complex than that of SZ patients. Effective treatment outcomes require the use of complex cognitive abilities and communication skills, including the comprehension of metaphors. Therefore, it is imperative to differentiate the high-level language skills of the two groups in order to be able to specifically tailor treatment strategies to compensate for different levels of metaphor comprehension impairment (42, 43).

Data about the relationship between the psychopathological symptoms of schizophrenia and metaphor comprehension is scarce, and analyses comparing the clinical pictures of SZ patients and SZ-SUD patients are inconclusive. Some reports suggest that patients with dual diagnoses have significantly more severe negative symptoms and general symptoms of schizophrenia (44)—they are more likely to exhibit symptoms of depression and anxiety in comparison to SZ patients (45, 46). Other reports suggest that individuals with SZ-SUD are characterized by fewer positive and negative symptoms when released from hospitalization than are SZ patients (47) or that they do not differ from patients with a single diagnosis in terms of symptom severity (46, 48, 49).

Therefore, this study aims to investigate the ability to comprehend conventional metaphors in SZ patients and SZ-SUD patients, comparing results with a healthy control group. Another goal is to estimate the relationship between the presence of psychopathological symptoms and the ability to understand metaphors in both clinical groups. We hypothesized that due to the additional burden of SUD, which may have neurotoxic effects, dual diagnosis patients would show greater impairment of the ability to understand metaphors. We also expected that there would be a relationship between psychopathological symptoms and comprehension of metaphors in SZ and SZ-SUD patients.

## Materials and Methods

### Participants

A total of 120 individuals took part in the study: 40 individuals diagnosed with paranoid schizophrenia (SZ; aged 19–58); 40 with paranoid schizophrenia and substance use disorder (SZ-SUD; aged 19–55); and 40 healthy controls (HC; aged 19–58) matched in terms of age, gender, and education. The diagnosis was based on ICD-10 criteria (50). In the SZ-SUD group, there were 18 patients with alcohol use disorder, 10 with drug use disorder (9 people using amphetamine and marijuana simultaneously, 1 person using amphetamine and cocaine), and 12 with both alcohol and drug use disorders (mainly amphetamine and marijuana simultaneously or amphetamine alone). SZ-SUD patients were selected based on medical history, consultation with psychiatrists, and a clinical interview based on the ICD-10 with the patient which was performed before the study. Due to the fact that the average duration of the substance use disorder was *M* = 9.65 (*SD* = 6.62) years, based on Adan et al. (27), we assumed that SUD leads to neurotoxicity in people with a schizophrenia spectrum disorder. Adan et al. (27) showed that neurobiological alterations can be seen in SZ-SUD patients after 5 years of illness or longer. Moreover, unlike clinical severity or specific schizophrenia diagnosis, SUD characteristics are important modulating factors. All patients were undergoing antipsychotic treatment and were clinically stable. Inclusion criteria were: being aged between 18 and 60, comprehension of the study procedures, and written consent to taking part in the study. Exclusion criteria were: neurological conditions, chronic somatic conditions, brain injury, intellectual disability, dementia, and, in the case of the patients, other psychiatric conditions. The study was conducted in psychiatric and therapeutic clinics. The study was approved by the Ethics Committee for Research Projects at the Institute of Psychology of the University of Gdańsk (6/2015).

### Clinical Assessments

The Positive and Negative Syndrome Scale [PANSS; (51)] was used to measure the severity of positive, negative, and general psychopathological symptoms in the clinical groups.

### Neuropsychological Assessment

The ability to understand and explain conventional metaphors was assessed by the four subtests of the Polish Version of the Right Hemisphere Language Battery (RHLB-PL): the Written Metaphor Test, the Written Metaphor Explanation Test, the Picture Metaphor Test, and the Picture Metaphor Explanation Test (5, 52).

The Picture Metaphor and Written Metaphor tests require the participant to select one answer (from several) corresponding to the correct meaning of a metaphor. Only one answer is correct. The other answers constitute either literal answers or “inappropriate meaning” type errors.

The Picture Metaphor Test is composed of a list of eleven statements including a metaphor accompanied by a set of four pictures. The participant must select the picture which represents the correct meaning of the metaphor. The first statement serves as an example to present the instructions and to make sure the participant has understood the instructions; the rest constitute tasks. The Written Metaphor Test also consists of 11 statements (1 example and 10 scored). Each task is on a separate page.

In the Picture Metaphor Explanation Test and the Written Metaphor Explanation Test, the participant has to explain the meaning of metaphors from the Picture Metaphor and Written Metaphor tests in their own words. Answers can be classified as correct, abstract incorrect, or literal incorrect.

On all of the aforementioned tests, a correct answer is worth one point and a maximum of 10 points can be scored on each test.

### Statistical Analysis

Statistical analysis of the results was done using the IBM Statistical Package for the Social Sciences (SPSS, version 24). The normality of the distribution was tested with the Shapiro-Wilk test. The differences among the three groups in terms of demographic variables (age and years of education) and neuropsychological variables (difference in incorrect metaphor comprehension answers) were examined with one-way analysis of variance (ANOVA). The Games-Howell test was used to examine *post hoc* differences among the subsamples because variances were not homogeneously distributed in each group. For multiple comparisons, Bonferroni correction was used. Student's *t* test was used to check for differences in psychopathological symptoms between the two clinical groups. To determine differences in correct answers on metaphor comprehension tests, we used a multivariate two-way repeated measures/mixed model multivariate ANOVA (MANOVA). Finally, in order to assess the relationship between intensity of psychopathological symptoms and metaphor comprehension in both schizophrenia groups, Pearson's *r* correlation coefficient was estimated. If there was a significant correlation, separate single stepwise linear regression procedures were conducted—one for each of the two groups.

## Results

### Demographic and Clinical Characteristics

Groups did not differ in terms of gender, age, or years of education. Moreover, there were no differences between the clinical groups either in terms of the severity of positive, negative, or general symptoms, or in terms of number of hospitalizations, time since diagnosis, or age at first hospitalization. Results are presented in **Table 1**.

**Table 1:** Descriptive demographic and clinical characteristics of patients with schizophrenia without (SZ) and with substance use disorder (SZ-SUD), as well as healthy controls (HC).

	SZ	SZ-SUD	HC	χ^2^/*F*/*t*	*p*
Gender: m/f	27/13	31/9	27/13	1.29^a^	0.524
Age in years: *M* (*SD*)	35.95 (9.76)	35.87 (8.91)	35.30 (10.31)	0.05^b^	0.948
Years of education: *M* (*SD*)	12.50 (2.93)	12.05 (2.63)	11.67 (1.89)	1.07^b^	0.347
Schizophrenia duration in years: *M* (*SD*)	11.78 (9.24)	11.09 (7.44)	–	−0.37^c^	0.712
SUD duration in years: *M* (*SD*)	–	9.65 (6.62)	–	–	–
Number of hospitalizations: *M* (*SD*)	9.22 (9.42)	10.77 (7.73)	–	0.80^c^	0.424
Age at first hospitalization: *M* (*SD*)	24.40 (6.68)	24.82 (7.64)	–	−0.80^c^	0.985
Positive symptoms (PANSS): *M* (*SD*)	15.94 (6.61)	14.80 (6.28)	–	−0.78^c^	0.438
Negative symptoms (PANSS): *M* (*SD*)	19.32 (9.95)	18.00 (7.99)	–	−0.65^c^	0.520
Global symptoms (PANSS): *M* (*SD*)	39.59 (14.46)	36.42 (11.90)	–	−1.05^c^	0.296

PANSS, Positive and Negative Syndrome Scale.

^a^Chi-squared test. ^b^One-way analysis of variance F test. ^c^Student's t test.

### Group Differences in Correct Answers on Metaphor Comprehension Tests

To test the effects of the type of metaphor tests or explanation tests, a multivariate two-way repeated measures/mixed 3 x 2 MANOVA with “group” (SZ patients *vs.* SZ-SUD patients *vs.* HC) as a between-subject factor and “test” as within-subject factor were computed separately for “type of metaphor” (Picture Metaphor Test *vs.* Written Metaphor Test) and “type of explanation” (Picture Metaphor Explanation *vs.* Written Metaphor Explanation).

**Figure 1:**
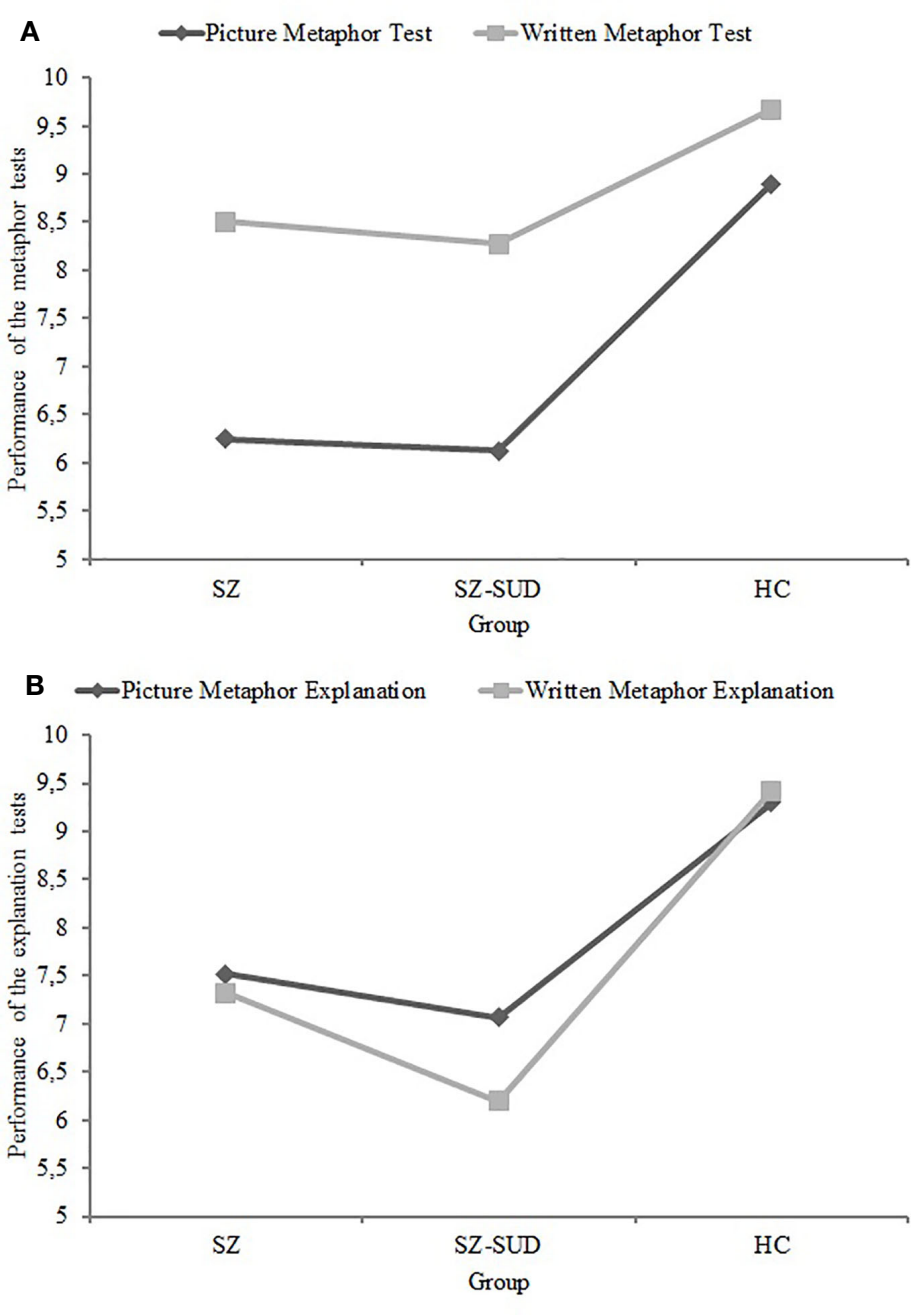
Mean scores in patients with schizophrenia without (SZ) and with (SZ-SUD) substance use disorder and healthy controls (HC) on metaphor tests **(A)** and on explanation tests **(B)**.

As can be seen in **Table 2** and **Figure 1A**, the main effect of “type of metaphor” was large and significant [*F*_(1,__117)_ = 74.08; *p* < 0.001; ɳ^2^ = 0.39], indicating that the differences in results between the two metaphor tests were significant across all participants. The main effect of “group” was also large and significant [*F*_(2,__117)_ = 20.04; *p* < 0.001; ɳ^2^ = 0.26], indicating that there were differences between the three groups in results on both metaphor tests. *Post hoc* analysis showed that SZ patients and SZ-SUD patients did not differ on either metaphor test, but they had lower results than healthy controls. Furthermore, the interaction between “type of metaphor” and “group” was large and significant [*F*_(2,__117)_ = 6.21; *p* = 0.003; ɳ^2^ = 0.10]. In the three groups, pairwise comparisons revealed that participants had scored better on the Written Metaphor Test than on the Picture Metaphor Test (0.05 > *p* < 0.001). Pairwise comparisons showed that SZ patients had worse results than HC on the Picture Metaphor Test (*p* < 0.001) and on the Written Metaphor Test (*p* < 0.05), and SZ-SUD patients had worse results on the Picture Metaphor Test (*p* < 0.001) and on the Written Metaphor Test (*p* < 0.01) compared to HC.

**Table 2:** Differences in correct and incorrect answers in comprehension of metaphors between patients with schizophrenia without (SZ) and with substance use disorder (SZ-SUD) and healthy controls (HC).

Metaphor test	SZ (*n* = 40) *M* (*SD*)	SZ-SUD (*n* = 40) *M* (*SD*)	HC (*n* = 40) *M* (*SD*)	*F*	*p*	ɳ^2^	SZ *vs.* SZ-SUD	SZ *vs.* HC	SZ-SUD *vs.* HC
Correct answers in comprehension of metaphors									
**Picture Metaphor Test**				16.29^a^ 74.08^b^ 20.04^c^	0.003 0.000 0.000	0.10 0.39 0.26	1.000	0.000	0.000
Correct metaphorical answers	6.25 (2.53)	6.12 (2.5)	8.9 (1.41)						
**Written Metaphor Test**									
Correct metaphorical answers	8.5 (2.0)	8.27 (2.37)	9.67 (0.65)						
**Picture Metaphor Explanation**				4.18^a^ 4.84^b^ 21.15^c^	0.018 0.030 0.000	0.07 0.04 0.27	0.211	0.000	0.000
Correct metaphorical answers	7.52 (2.33)	7.07 (2.36)	9.3 (0.88)						
**Written Metaphor Explanation**									
Correct metaphorical answers	7.32 (2.47)	6.2 (2.7)	9.42 (0.87)						
Incorrect answers in comprehension of metaphors									
**Picture Metaphor Test**									
Literal answers	2.95 (2.20)	2.9 (2.20)	0.52 (0.9)	21.85^d^	0.000	0.27	0.994	0.000	0.000
Inappropriate meaning	1.02 (1.42)	0.97 (1.27)	0.57 (1.0)	1.57^d^	0.213	–	–	–	–
**Written Metaphor Test**									
Literal answers	0.62 (1.14)	0.67 (1.30)	0.0 (0.0)	5.60^d^	0.005	0.09	0.982	0.004	0.006
Inappropriate meaning	0.87 (1.38)	1.05 (1.5)	0.3 (0.6)	4.08^d^	0.019	0.07	0.851	0.049	0.014
**Picture Metaphor Explanation**									
Literal incorrect	0.45 (1.17)	0.5 (1.01)	0.1 (0.3)	2.28^d^	0.107	–	–	–	–
Abstract incorrect	1.97 (1.77)	2.2 (1.91)	0.6 (0.77)	12.15^d^	0.000	0.17	0.849	0.000	0.000
Lack of answer	0.05 (0.22)	0.22 (0.69)	0.0 (0.0)	3.13^d^	0.051	–	–	–	–
**Written Metaphor Explanation**									
Literal incorrect	0.47 (1.28)	0.77 (1.64)	0.05 (0.22)	3.64^d^	0.029	0.06	0.635	0.109	0.022
Abstract incorrect	2.02 (1.79)	2.65 (2.31)	0.52 (0.78)	15.59^d^	0.000	0.21	0.372	0.000	0.000
Lack of answer	0.17 (0.81)	0.32 (0.82)	0.0 (0.0)	2.36^d^	0.099	–	–	–	–

MANOVA model: ^a^F test of the interaction effect between “group” and “type of metaphor” or “type of explanation”. ^b^F test of the main effect of “type of metaphor” or “type of explanation”. ^c^F test of the main effect of “group”.

ANOVA model: ^d^F test of the main effect of “group”.

Models for the type of metaphor and the type of explanation were tested separately.

Moreover, as can be seen in **Table 2** and **Figure 1B**, the main effect of “type of explanation” was medium and significant [*F*_(1,__117)_ = 4.84; *p* = 0.030; ɳ^2^ = 0.04], indicating that the differences in results between the two explanation tests occurred across all participants. The main effect of “group” was large and significant [*F*_(2,__117)_ = 21.15; *p* < 0.001; ɳ^2^ = 0.27], indicating that there were differences between the three groups in results on the two explanation tests. *Post hoc* analysis showed that SZ patients and SZ-SUD patients did not differ on the two explanation tests, but they had worse results than healthy controls. Furthermore, the interaction between “type of explanation” and “group” was medium and significant [*F*_(2,__117)_ = 4.18; *p* = 0.018; ɳ^2^ = 0.07]. Pairwise comparisons revealed that only SZ-SUD patients had better results on Picture Metaphor Explanation than on Written Metaphor Explanation (*p* = 0.001). Pairwise comparisons showed that SZ patients had worse results on Picture Metaphor Explanation (*p* < 0.001) and on Written Metaphor Explanation (*p* < 0.001) than HC, and SZ-SUD patients had worse results on Picture Metaphor Explanation (*p* < 0.001) and on Written Metaphor Explanation (*p* < 0.001) compared to HC.

### Group Differences in Incorrect Answers in Metaphor Comprehension

As shown in **Table 2**, analysis revealed significant differences in errors in metaphor comprehension in all groups (0.029 > *p* < 0.001), except for inappropriate meaning on the Picture Metaphor Test, literal incorrect answers and lack of answer for Picture Metaphor Explanation, and lack of answer for Written Metaphor Explanation. The effect size (ɳ^2^) of comprehension of metaphor dysfunctions in schizophrenia was found to be 0.06–0.27, i.e., a small to large effect size. The results of *post hoc* analysis regarding differences in incorrect answers showed that SZ and SZ-SUD patients obtained a significantly higher number of literal incorrect answers on the Written (0.006 > *p* < 0.004) and Picture (*p* < 0.001) Metaphor tests, a higher number of abstract incorrect answers on Written (*p* < 0.001) and Picture (*p* < 0.001) Metaphor Explanation, and more inappropriate meanings on the Written Metaphor test (0.049 > *p* < 0.014) than did the HC group. Moreover, only the SZ-SUD group obtained higher scores for literal incorrect answers on the Written Metaphor Explanation Test (*p* < 0.022) than did the HC group. No differences were found between SZ and SZ-SUD groups on all measures.

### Relationship Between Psychopathological Symptoms and Comprehension of Metaphors

**Table 3** shows correlations between PANSS scores and metaphor processing tests in SZ patients. In this group, negative symptoms were significant predictors of the two indicators of the Picture Metaphor Test—correct metaphorical answers (β = −0.35; *t* = −2.21; *p* = 0.030; model was statistically significant, *F* = 4.88; *p* = 0.034) and literal answers (β = 0.33; *t* = 2.06; *p* = 0.047; model was statistically significant, *F* = 4.25; *p* = 0.047)—and of the two indicators of Written Metaphor Explanation—correct metaphorical answers (β = −0.37; *t* = −2.35; *p* = 0.24; model was statistically significant, *F* = 5.54; *p* = 0.024) and abstract incorrect answers (β = 0.34; *t* = 2.12; *p* = 0.041; model was statistically significant, *F* = 4.49; *p* = 0.041). About 10, 8, 11, and 9% of variance of these indicators was predicted by negative symptoms, respectively. Furthermore, in this clinical group, global symptoms were significant predictors of the two indicators of the Picture Metaphor test—correct metaphorical answers (β = −0.39; *t* = −2.52; *p* = 0.016; model was statistically significant, *F* = 6.37; *p* = 0.016) and literal answers (β = 0.37; *t* = 2.35; *p* = 0.025; model was statistically significant, *F* = 5.51; *p* = 0.025). About 13 and 11% of the variance of these indicators was predicted by global symptoms, respectively.

**Table 3:** Relationship between metaphor comprehension and psychopathological symptoms in schizophrenia patients without (SZ) and with substance use disorder (SZ-SUD).

Metaphor test	Psychopathological symptoms (in PANSS)					
	Positive	Negative	Global	Positive	Negative	Global
	SZ (*n* = 40)			SZ-SUD (*n* = 40)		
**Picture Metaphor Test**						
Correct metaphorical answers	−0.25	−0.35*	−0.39*	0.23	0.08	0.04
Literal answers	0.27	0.33*	0.37*	−0.13	−0.13	−0.15
Inappropriate meaning	0.02	0.11	0.12	−0.22	0.06	0.18
**Written Metaphor Test**						
Correct metaphorical answers	−0.13	−0.15	−0.17	−0.13	−0.08	−0.11
Literal answers	0.21	0.21	0.22	0.03	−0.03	0.04
Inappropriate meaning	0.01	0.04	0.06	0.18	0.16	0.15
**Picture Metaphor Explanation**						
Correct metaphorical answers	−0.08	−0.17	−0.02	0.07	−0.18	−0.13
Literal incorrect	0.15	0.14	0.06	−0.17	0.02	0.07
Abstract incorrect	0.00	0.12	−0.01	0.08	0.17	0.08
Lack of answer	−0.02	0.08	−0.04	−0.21	0.10	0.12
**Written Metaphor Explanation**						
Correct metaphorical answers	−0.22	−0.37*	−0.31	−0.12	−0.35*	−0.29
Literal incorrect	0.09	0.11	0.09	−0.05	0.03	0.08
Abstract incorrect	0.17	0.34*	0.32	0.19	0.41**	0.25
Lack of answer	0.14	0.20	0.08	−0.06	−0.04	0.07

PANSS, Positive and Negative Syndrome Scale.

*p < 0.05. **p < 0.01.

**Table 3** shows correlations between PANSS scores and metaphor processing tests in SZ-SUD patients. In this group, negative symptoms were significant predictors of the two indicators of Written Metaphor Explanation—correct metaphorical answers (β = −0.35; *t* = −2.31; *p* = 0.027; model was statistically significant, *F* = 5.31; *p* = 0.027) and abstract incorrect answers (β = 0.41; *t* = 2.74; *p* = 0.009; model was statistically significant, *F* = 7.49; *p* = 0.009). About 10 and 14% of the variance of these indicators was predicted by negative symptoms, respectively.

In both groups, no significant predictor was identified for any indicators of metaphor processing tests.

## Discussion

This study examined differences in metaphor comprehension between healthy controls and SZ patients and SZ-SUD patients. Regardless of SUD status, the schizophrenia patients showed an impaired ability to interpret and explain metaphors. These results are in-line with previous research using other measurement tools for the assessment of metaphor processing (12, 14, 53, 54). Our results are similar to the findings of Bambini et al. (55), which revealed that SZ patients have difficulty with three types of figurative language tasks: idioms, metaphors, and proverbs. Moreover, similar results were previously reported by Mo et al. (6), who showed that SZ patients make incorrect interpretations and do not understand non-literal meanings; they also found that this holds for patients in remission and that results do not change when controlling for IQ (5). Mossaheb et al. (16) also found a decreased ability to paraphrase and identify appropriate metaphors (both novel and conventional). Additionally, our results were partially in-line with research by Pawełczyk et al. (15), who showed that SZ patients had difficulty explaining metaphors in their own words and using their general knowledge. However, in contrast to our study, participants correctly selected the meaning of written metaphors from the three possible answers and were able to identify the appropriate picture illustrating the meaning of a metaphor. These differences between the studies may be because the patients in our study were older (by an average of 10 years) and had suffered from the disease for longer (by an average of 8 years), which could have contributed to greater difficulties processing metaphors. Deficits manifested as poorer processing of metaphorical content in individuals with SZ may be an effect of impaired functioning of the brain (13, 19).

It is interesting that, in this study, participants from all groups scored lower on visual metaphor comprehension than on verbal metaphor comprehension. One possible explanation of this is that, after verbal instruction, the visual mental images evoked by the figurative expressions might become somewhat intrusive in the visual metaphor task. Therefore mental images generated by a figurative expression may make it harder to select the correct metaphorical answer based on pictorial material. SZ patients and SZ-SUD patients might be especially challenged by figurative language and by this shift from mental images to pictorial answers, given their tendency toward concrete thought and their inability to think beyond the immediate aspects of the stimuli (55, 56).

In the current study, SZ patients and SZ-SUD patients gave significantly more literal and abstract inappropriate answers than did the healthy controls, which is in-line with previous studies (10, 15). Moreover, research by Chapman (3) and Elvevag et al. (57) showed that SZ patients are more likely to give incorrect literal answers in comparison to healthy individuals. Incorrect interpretations made by patients may be caused by impaired inferences about reality and impaired processing of various types of content. Inappropriate information processing is most likely associated with excessive generalization or concentration on concrete stimuli and inaccurate identification of significant information (58). Differences in terms of the amount of errors made by patients and healthy individuals may stem from deficits in language functions and abstract thinking in schizophrenia patients.

The results of this study failed to support the hypothesis that those with dual diagnoses would have greater difficulty with metaphor comprehension than would SZ patients. It may be that the detrimental effects of the substances in question on the brain were not strong enough to cause significant additional decline in the ability of understanding metaphors, which is already impaired in schizophrenia (27). Only SZ-SUD patients had greater difficulty verbally expressing the meanings of written metaphors than picture metaphors. These tasks required the patients to use their general knowledge and memory of the information encoded in the previous test (The Written Metaphor Test or Picture Metaphor Test). The pictures from the Picture Metaphor Test could have made the task easier by allowing for better memorization and explanation of figurative meanings than did the text from the Written Metaphor Test. To the best of our knowledge, no other study has investigated the processing of metaphors in people with SZ-SUD, thus it was impossible to compare the results of the current study to the works of other authors. Despite a lack of data regarding metaphor comprehension, some reports suggest that the fitness of cognitive functions such as verbal fluency, motor speed, memory, executive functions, information processing, and verbal abilities are similar between SZ patients and SZ-SUD patients (42, 59–62). Another potential explanation of the lack of differences in the studied processes may be the similar severity of the psychopathological symptoms of schizophrenia. However, this requires further research.

Patients in the two clinical groups did not differ in terms of the average number of concrete or abstract errors. SZ-SUD patients gave more literal incorrect answers than did the HC group on the Written Metaphor Explanation Test, while there were no differences between the HC and SZ patients. Moreover, only SZ-SUD patients had lower scores on the Written Metaphor Explanation Test than on the Picture Metaphor Explanation Test. One possible reason for this could be that the metaphor explanation tests were preceded by written and pictorial metaphor tests, which do not involve similar mental processes. SZ-SUD patients performed worse when the verbal explanation test was preceded by a test also based on verbal material than when preceded by a test based on non-verbal (pictorial) material. The open response format of the Written Metaphor Explanation Test requires executive and verbal expressive abilities for planning and articulating the response, which may be additionally impaired by the presence of a substance use disorder (63) and could have made subjects more susceptible to making concrete errors (55). Because there is not enough data in this context, further research on the importance of executive functions and speech planning for metaphor processing in schizophrenia with substance use disorder should be conducted. The observed differences in errors made by groups may be caused by impaired executive functions, working memory, or theory of mind (1, 6, 64). Moreover, errors made by patients may also stem from poverty of speech (65), which is associated with impaired ability to hold contextual information, faulty goal orientation, and speech disorganization (66).

Furthermore, the current study showed that greater severity of negative symptoms is associated with decreased ability to explain written metaphors in SZ patients and SZ-SUD patients as well as with greater difficulty understanding visual metaphors in SZ patients. The severity of negative and global symptoms was also associated with a higher number of errors. Mossaheb et al. (16) also showed that severity of negative symptoms is associated with metaphor comprehension. On the other hand, a relationship between the severity of schizophrenia symptoms and metaphor processing was not confirmed by Pawełczyk et al. (15) in a study on the relationship in SZ patients between understanding and explaining written and visual metaphors. Some of the differences between our results and the results of Pawełczyk et al. (15) may stem from the socio-demographic and illness duration differences between the studied groups. However, the relationship between metaphor comprehension and symptoms of schizophrenia requires further analysis. It is worth mentioning that a high severity of negative symptoms fosters deficits in cognitive functions, which has been shown in numerous studies (67). On the other hand, in individuals with a dual diagnosis, there is a complex relationship between substance use, executive functions, and psychopathology. Substance use may be a confounding factor, and it may mask the relationship between negative symptoms and cognitive functioning (68). It could therefore be expected that there is also a complex relationship between these factors and visual metaphor processing.

The ability to process metaphors plays an important role in the correct perception of reality. Impairments understanding and explaining metaphors may lead to difficulties in the everyday functioning of not only the patients themselves, but also their families, through lack of understanding of reality, social situations, and the sense of being misunderstood. Gathering detailed knowledge about the specifics of impairments in figurative content processing, assessing these systematically at different stages of schizophrenia, as well as investigating their relations with other cognitive functions and clinical factors may be important for clinical diagnostics. Moreover, studying the various errors made by patients may allow for a better understanding of their way of thinking (10). Metaphors are central to communication: they convey social and affective information and can potentially influence both reasoning and decision making (69, 70). Therefore we might infer that metaphor comprehension affects social functioning—a central part of quality of life as assessed by the Quality of Life Scale (QLS). While the variance was only partially explained, we consider this finding very important, as it provides a direction for further investigation and for a deeper examination of the role of communication skills in quality of life and as a target for intervention (55). Including training of metaphor-processing skills in the therapeutic process may provide new opportunities for improving patients' quality of life. Moreover, a better understanding of the cognitive profile of individuals with SZ-SUD may help adjust treatments to their needs and to improve their cognitive and communication abilities.

Interpretation of these findings should take into account the limitations of this study. Individuals with SZ-SUD used various psychoactive substances—the specifics of these substances were not analyzed and require further investigation. Information regarding the quantity of used substances was not controlled, but it should be noted that such variables are very hard to verify when relying on interviews with patients (38, 71). The neurotoxic effect was not measured; however, based on Adan et al. (27), we assumed that neurobiological alterations would be seen in SZ-SUD patients after 5 or more years of illness. Moreover, craving scales or consumption scales were not used to quantify the severity of the substance use disorder. IQ levels were also not controlled. However, it is not certain that IQ levels are associated with the ability to process metaphors, because results are inconclusive in this regard (6, 17, 72, 73). The effect of the patients' antipsychotic medications (equivalent chlorpromazine) on metaphor processing skills or psychopathological symptoms was not examined in either group. However, previous research suggests no correlation between the dosage of antipsychotic medication and patients' abilities to understand metaphors and irony (6). Another limitation is that we did not use the Clinical Global Impression-Severity (74) to measure stability criteria in the clinical groups.

## Data Availability Statement

The datasets generated for this study are available on request to the corresponding author.

## Ethics Statement

The studies involving human participants were reviewed and approved by Ethics Committee for Research Projects at the Institute of Psychology of the University of Gdańsk (6/2015). The patients/participants provided their written informed consent to participate in this study.

## Author Contributions

All authors contributed to and have approved the final manuscript: “Metaphor Processing Dysfunctions in Schizophrenia Patients With and Without Substance Use Disorders.” EK was the principal coordinator of the project, was involved in the study design, took part in recruitment of the patients, conducted research, managed the literature searches and analyses, wrote the first draft of the manuscript, and undertook the statistical analysis. ET managed the literature searches and analyses, undertook the statistical analysis, wrote the first draft of the manuscript, and recruited participants. KK undertook the statistical analysis and corrected the manuscript. AS corrected the manuscript. MM corrected the manuscript. MF-Ż corrected the manuscript. WR was involved in the study design and corrected the manuscript.

## Conflict of Interest

The authors declare that the research was conducted in the absence of any commercial or financial relationships that could be construed as a potential conflict of interest
